# Sex differences in the serum level of endogenous ligands for estrogen receptor β in the elderly population

**DOI:** 10.1038/srep25878

**Published:** 2016-05-11

**Authors:** Miyuki Kobayashi, Nobuhiro Sugiyama, Daimei Sasayama, Hidehiko Sasamoto, Yoshimichi Miyashiro, Kunimasa Arima, Shinsuke Washizuka

**Affiliations:** 1 Department of Psychiatry, Shinshu University School of Medicine, 3-1-1 Asahi, Matsumoto, Nagano 390-8621, Japan; 2 Department of Psychiatry, National Hospital Organization Komoro Kogen Hospital, 4598 Ko, Komoro, Nagano 384-8540, Japan; 3 ASKA Pharmaceutical Medical Co., Ltd. 5-36-1 Shimosakunobe, Kawasaki Takatsu-ku, Kanagawa 213-8522, Japan

## Abstract

Animal studies suggest that estrogen receptor β (ERβ)-agonists, but not ERα-agonists, are antidepressants. Several endogenous ligands for ERβ have been proposed, including 5α-androstane-3β, 17β-diol (3βAdiol), Androstenediol (Δ5-diol), and 7α-hydroxydehydroepiandrosterone (7α-OH-DHEA). The aim of this study was to determine the serum and salivary levels of natural ERβ ligands in men and women with and without past depressive episodes in the elderly population. DHEA (a precursor of 3βAdiol, Δ5-diol, and 7α-OH-DHEA), 17β-estradiol (E2), and cortisol (F) were also measured. Samples were collected from 51 subjects and liquid chromatography tandem mass spectrometry was used for measurement. Comparisons were made between groups based on sex and depression history. E2, 3βAdiol, and Δ5-diol levels were significantly lower in women than in men regardless of depression history. There were no significant differences between men and women in DHEA or 7α-OH-DHEA levels. DHEA was significantly lower in women with depression than in women without depression. Reduced DHEA levels may be related to depression vulnerability in women. Further studies are needed to determine the mechanism underlying sex differences in the prevalence of depression and increased risk of depression during menopause. Not only E2 but also two other estrogenic steroids (3βAdiol and Δ5-diol) should be involved in these studies.

Our previous animal experiment has shown the following: [1] Estrogen receptor β (ERβ), not estrogen receptor α (ERα), is abundantly expressed in the lateral part of the dorsal raphe (DR); [2] ovariectomy (OVX) leads to the loss of ERβ and loss of tryptophan hydroxylase- (TPH-) immunoreactivity in this area of the midbrain; and [3] TPH levels can be restored by the administration of 17β-estradiol (E2) and, more strongly, by an ERβ-selective ligand (LY3201) within a critical temporal window after OVX^1^. Similarly, Donner *et al*. have shown that administration of another ERβ-selective ligand (diarylproprionitrile) increases *Tph2* mRNA expression in female rat brain after OVX and that it decreases their depression-like behavior in the forced swim test paradigm^2^. These results suggest that ERβ mediates estrogen regulation of TPH levels and that ERβ ligands may be useful for the prevention of postmenopausal depression if administered shortly after menopause. However, it is still unknown which endogenous ERβ ligands are related to the pathophysiology of depression.

Several endogenous ligands of ERβ have been proposed. Of these, 5α-androstane-3β, 17β-diol (3βAdiol) has been studied most extensively^3^. 3βAdiol is an estrogenic metabolite of 5α-dihydrotestosterone (DHT)^4^. The binding affinity of 3βAdiol is stronger for ERβ than for ERα^5^. In HT-22 neuronal cells, 3βAdiol activates ERβ-induced transcription mediated by an estrogen response element with the same efficacy as E2, and the transcriptional selectivity for ERβ over ERα is much greater than the binding selectivity in these neuronal cells^6^. Several animal experiment paradigms suggest that 3βAdiol is anxiety reducing, cognitive enhancing^7^, and anti-depressive^8^ [reviewed in^9^]. Another endogenous ligand of ERβ is Androstenediol (Δ5-diol), which is synthesized from dehydroepiandrosterone (DHEA) without the need for 5α-reductase. The binding affinity of Δ5-diol is stronger for ERβ than for ERα, and is similar to that of 3βAdiol^5^. Δ5-diol prevents activation of microglia^10^. DHEA is a precursor of both 3βAdiol and Δ5-diol (Fig. 1)^11^. Another ligand generated from DHEA by cytochrome P450, family 7, subfamily B, polypeptide 1 (CYP7B1) is 7α-hydroxy-dehydroepiandrosterone (7α-OH-DHEA), which is an ERβ activator^12^,^13^. Although 7α-OH-DHEA is a less efficient ERβ activator than 3βAdiol^12^,^13^, it is a candidate natural ligand for ERβ. In humans, the concentration of 7α-OH-DHEA in serum is strongly correlated with that in cerebrospinal fluid^14^. In this study, we investigated the pathway from DHEA to 3βAdiol, Δ5-diol, and 7α-OH-DHEA in terms of the “DHEA-ERβ axis”.

Depression is much more common in women than in men^15^,^16^ and the risk of depression increases during menopause^17^. The DHEA-ERβ axis may be involved in the etiophysiology of depression, especially in geriatric women.

We determined the serum and salivary levels of natural ERβ ligands in men and women with and without past depressive episodes. We focused on geriatric subjects close to the peak age of depression onset. In addition to an interview regarding menopause, we measured E2 levels in women. We measured cortisol (F) levels as a marker of general stress (For chemical structures of the steroids of interest, please see Supplementary Figure S1). We collected both saliva and serum to investigate the feasibility of estimating the serum levels of steroids without blood sampling. This preliminary study has generated the first data on the precise levels of endogenous ERβ ligands in geriatric subjects, as determined using liquid chromatography tandem mass spectrometry (LC-MS/MS) technology.

## Results

### Clinical Data

The scores of psychiatric inventories confirmed the exclusion of subjects with an acute phase of depression, dementia, or mild cognitive impairment. No subject had a history of oophorectomy or orchidectomy. There was a significant difference in age of subjects between female controls and the female depression group (p < 0.01, Dunn’s test). The homogeneity of variance was confirmed for BMI data (p = 0.6999, Bartlett’s test), and there were no significant differences between the groups (p = 0.1609, one-way ANOVA). There was no significant difference in body fat percentage or abdominal circumference between the control and depression groups of either sex (Welch’s test). (Table 1).

### Serum Levels of Steroids

There were no sex differences in the serum levels of DHEA and 7α-OH-DHEA either in control or depression subjects. However, there were statistically significant differences between sexes in the levels of 3βAdiol, Δ5-diol, and E2; males exhibited higher levels of these three steroids than females. The female control group had a higher level of DHEA than the female depression group. Because there was a significant difference in age between these two groups, Spearman correlation coefficients were used to evaluate the correlation between serum levels of DHEA and age; there were no significant correlations. The other steroids measured in this study did not differ between control and depression subjects. Male depression subjects had a higher level of F than female depression group (Table 2, Supplementary Figure S2).

### Correlation between the Serum and Salivary Levels of Each Steroid

For DHEA, 7α-OH-DHEA, and F, strong correlations were observed between serum and salivary levels. There were also correlations between serum and salivary levels of 3βAdiol; however, most of the measured values of 3βAdiol in saliva were under the quantitation limit. The measured values of Δ5-diol in both saliva and serum were within the quantitation limit, but there were no significant correlations between them. We failed to confirm a correlation between the levels of E2 in saliva and serum (Table 3, Supplementary Figure S3).

## Discussion

It has been well established that the serum level of E2 is lower in women after menopause, and this may explain the high prevalence of depression in postmenopausal women. In contrast, elderly men do not have such an acute, dynamic change of endocrine system that occurs in women^18^,^19^,^20^. This could be one explanation of why depression is more common in women. Our data on serum E2 concentration agree well with other reports both in elderly men^21^,^22^ and postmenopausal women^23^. The lower level of E2 in postmenopausal women than those in men might reflect that the production of E2 in women is highly dependent on ovarian function, whereas men produce E2 using the different pathway^21^,^22^.

In this study, we showed that not only levels of E2 were lower in women, both with and without depression, but levels of 3βAdiol and Δ5-diol were also reduced. Interestingly, there was no sex difference in the level of DHEA, the precursor of these steroids. Levels of 7α-OH-DHEA, a metabolite of DHEA with a weak affinity for ERβ, also did not differ between sexes. Both men and women with depression in this study were taking antidepressants as maintenance therapy, but showed the same trend in sex differences as in the control group. This suggests that sex difference is not a direct effect of antidepressant medication.

It is controversial whether hormone-replacement therapy (HRT) helps to keep women healthy after menopause. A large-scale randomized controlled study, the Women’s Health Initiative (WHI), failed to show the benefit of HRT^24^. WHI sub-analysis by the age groups at which HRT was started suggests that HRT timing is important (timing hypothesis or critical window theory); if HRT is started early, it may decrease the risk of cardiovascular disease^25^. Our previous animal model of postmenopausal depression also suggested a critical temporal window for hormone replacement^1^. However, the Kronos Early Estrogen Prevention Study (KEEPS), in which HRT was started within 3 years after menopause, revealed no difference between the treatment group and the placebo group in the annual change of carotid artery intima-media thickness. Here, we propose that not only E2, but also two other estrogenic steroids with an affinity for ERβ, 3βAdiol and Δ5-diol, are important for HRT. According to the yin-yang hypothesis of ERs^26^, activation of ERα is anxiogenic and that of ERβ is anxiolytic and anti-depressive. This has been repeatedly confirmed by animal experiments^26^. In our model, LY3201 (an ERβ-specific agonist) was more effective than E2 in maintaining high levels of TPH-positive neurons^1^. If an ERβ-specific agonist is used for HRT within a critical temporal window instead of E2, HRT should result in decreased incidence and severity of postmenopausal depression. Negative data from the WHI and KEEPS suggest that timely E2 replacement is not enough. Because we are now aware of steroid metabolites that can selectively activate ERβ, it is imperative that we investigate the role of these steroids in depression.

In order to rigorously test the relationship between ERβ ligands and postmenopausal depression, we need to determine the levels of endogenous ERβ ligands in younger subjects and whether pre-menopausal women and men have similar ERβ-ligand levels. Although they used a different measurement method with limitations, Habrioux *et al*. studied serum 3βAdiol levels of men and women aged 18 to 30 years^27^; the level in men was higher than that in women, but the sexual difference was smaller than that observed for the older individuals in our study.

In the present study, there were no differences between depressed patients and controls in the levels of 3βAdiol or Δ5-diol. This was an unexpected result, as we assumed that normal healthy individuals without a history of depression had relatively high levels of these ERβ ligands. This might be because subjects in the depression group were fully remittent. If we were to collect samples during the acute phase of depression, differences in the levels of these ligands might be observed between groups.

As in earlier studies^28^,^29^, women in the present study with a history of depression had significantly lower levels of DHEA than control women. It remains difficult to connect lower levels of DHEA to the mechanism of postmenopausal depression. In this study, all depression subjects were fully remittent and exhibited no difference from controls in aspects of daily life. Moreover, although the average serum level of DHEA in female depression subjects was lower than that in controls, it was 1.50 ng/mL. This is a very high concentration in comparison to other neuroactive steroids. Finally, there is no known specific controller of, or receptor for, DHEA. Therefore, we agree with recommendations of the Endocrine Society in 2014 that DHEA therapy should not be administered, because too many questions remain to justify its use^30^.

It seems unlikely that a lack of DHEA itself is responsible for depression in postmenopausal women. Instead, we assume that a high level of DHEA may indirectly reflect a strong DHEA-ERβ axis. Women with a strong DHEA-ERβ axis can generate 3βAdiol and Δ5-diol from DHEA quickly, as needed to prevent acute phases of depression. An *in vitro* study suggested that high DHEA levels strongly suppress CYP7B1-mediated 3βAdiol metabolism, resulting in high 3βAdiol levels and increased ERβ activation^13^. To determine whether a strong DHEA-ERβ axis enables female patients to avoid depressive episode, a large-scale, population-based prospective cohort study measuring not only DHEA, but also 3βAdiol and Δ5-diol, is needed. If we are correct, DHEA could be used as a reliable marker for vulnerability to postmenopausal depression.

Identifying the factors that control production of 3βAdiol and Δ5-diol from DHEA is also of great importance in understanding the mechanism of estrogen-related depression. The duration of action of 3βAdiol is determined by the concentration of CYP7B1 in the cells. This pathway from DHEA to effective levels of an ERβ ligand occurs within target cells and plasma levels of 3βAdiol do not reflect what is occurring in the ERβ target cells. Much more effort is needed to understand how the component enzymes in this pathway are regulated and which of the steps are defective when, despite high levels of DHEA, insufficient 3βAdiol is produced.

We must pay meticulous attention to minimize stress on subjects while collecting samples, especially from subjects with depression. In this study, we excluded patients in the acute phase of depression, and only recruited remittent patients in maintenance therapy. Rather than collect samples at multiple times throughout the day to check for circadian variation, we collected samples from all subjects at the same time of day. We also aimed to estimate the serum level of steroids using saliva without the need to draw blood. To collect blood or CSF, subjects must come to the hospital at a scheduled time and experience pain during collection. In contrast, saliva can be collected by subjects at home as many times as needed with minimal stress. In this study, we did not find convicting results showing correlation between salivary and serum levels of E2, 3βAdiol, or Δ5-diol. However, we believe using saliva instead of blood is valuable for future projects and we hope to improve our methodology.

## Materials and Methods

### Study Subjects

This study was approved by the Ethics Committee of Shinhu University School of Medicine (No. 1839) and the National Hospital Organization of Komoro Kogen Hospital. Due to ethical issues regarding depression, we included only fully remitted patients in this study. This study was conducted in accordance with the principles of the Declaration of Helsinki. All participants provided written informed consent.

Between 2011 and 2014, a total of 51 subjects, 65.2 ± 6.52 years of age (mean ± standard deviation), participated and were included in one of four groups: [1] nonclinical healthy geriatric males without any history of psychiatric or neurological disease (male control, n = 12, 65.7 ± 6.01); [2] female control (n = 16, 60.6 ± 2.42); [3] male subjects with a diagnosis of major depressive disorder who were in full remission, meaning that they had at least one previous depressive episode, but were normal with medical care at the time of the study (male depression, n = 10, 67.4 ± 7.38); and [4] female depression (n = 13, 68.8 ± 6.95). Subjects in groups [3] and [4] were receiving outpatient care. All patients had been diagnosed by trained psychiatrists, and 18 of 23 were being treated with antidepressants at the time of the sampling (5 with paroxetine, 5 with sertraline, 4 with duloxetine, 2 with mirtazapine, 1 with escitalopram, and 1 with amitriptyline). Before enrollment in the study, one of the authors (M.K.), a trained psychiatrist, confirmed all diagnoses using the Diagnostic and Statistical Manual of Mental Disorders-IV-TR. The inclusion criteria were as follows: All participants were (a) aged over 55 and for women, they were after menopause, (b) able to give informed consent and (c) physically and endocrinologically healthy at the time of this study. For group [1] and [2]: (a), (b), (c), and (d) no history of psychiatric disorder in the past. For group [3] and [4]: (a), (b), (c) and (e) Diagnostic and Statistical Manual of Mental Disorder-IV-TR diagnosis of major depressive disorder, in full remission. Exclusion criteria are as follows: (a) severe general medical condition, (b) dementing illness or mild cognitive impairment, (c) other conditions that would affect the results of this study, such as daily use of estrogenic drugs or under the anti-estrogen therapy.

### Clinical Data and Sample Collection

Anthropometric measurements (height, weight, body mass index [BMI], body fat percentage, abdominal circumference) and psychiatric inventories (17-item Hamilton Rating Scale for Depression [HAM-D], Beck Depression Inventory II [BeckII], Clinical Dementia Rating [CDR], Mini-Mental State Examination [MMSE], and Frontal Assessment Battery [FAB]) were conducted by a single trained psychiatrist (M.K.) at the time depression diagnoses were confirmed.

To avoid circadian variation of steroids levels, we collected samples from all subjects at the same time of day (10:00 a.m.). Blood was drawn while subjects were resting. Before collecting saliva, subjects rinsed their mouths with tap water. Separated serum and saliva were stored at −20 °C until they were processed for steroid measurement.

### Steroid Measurement

#### Materials and Reagents

F, DHEA, Δ5-diol, E2, and fusaric acid (FA) were from Sigma-Aldrich (St. Louis, MO, USA). 7α-OH-DHEA and 3βAdiol were from Steraloids (Newport, RI, USA). F-d_4_ was from C/D/N Isotopes Inc. (Quebec, Canada). DHEA-^13^C_3_ was synthesized at Tohoku Pharmaceutical University (Miyagi, Japan). Δ5-diol-d_4_ and 7α-OH-DHEA-d_2_ were synthesized at ASKA Pharma Medical Co. Ltd. (Kanagawa, Japan). E2-^13^C_4_ was from Hayashi Pure Chemical Ind. Ltd. (Osaka, Japan). 4-dimethylaminopyridine (DMAP), 2-methyl-6-nitrobenzoic anhydride (MNBA), and picolinic acid (PA) were from Tokyo Chemical Industry (Tokyo, Japan). Triethylamine (TEA), *O*-ethylhydroxyl-ammonium chloride, pentafluoropyridine, and 1 M sodium hydroxide solutions were from Wako Pure Chemical Industries (Osaka, Japan). OASIS MAX cartridge (60 mg, 3 mL) was from Waters Corporation (Milford, MA, USA). InertSep SI cartridge (500 mg, 3 mL) was from GL Science (Tokyo, Japan). All other reagents and solvents were of analytical grade.

#### Extraction and Purification

As internal standards, F-d_4_, DHEA-^13^C_3_, Δ5-diol-d_4_, 7α-OH-DHEA-d_2_, and E2-^13^C_4_ were added to serum and saliva samples. Steroids were extracted with ethyl acetate. After the organic layer was evaporated to dryness, the extract was cleaned with an Oasis MAX cartridge. After purification, the extract was evaporated to dryness, and each steroid was then subjected to derivatization.

#### Derivatization and Application to LC-MS/MS

##### F, DHEA, Δ5-diol, and 3βAdiol

The residue was treated with 50 μL reagent mixture (MNBA, 80 mg; PA, 40 mg; DMAP, 20 mg in 1 mL acetonitrile) and 10 μL TEA, and then left at room temperature for 30 min. After derivatization, the reaction mixture was purified with an InterSep SI cartridge. After purification, the extract was evaporated to dryness, the residue was dissolved in acetonitrile/distilled water (40:60, v/v), and the solution was subjected to LC-MS/MS. For quantitation, the *m/z* transitions 468.2 → 309.2, 472.2 → 450.3, 394.3 → 175.1, 397.4 → 178.4, 501.3 → 255.4, 503.3 → 257.1, and 505.4 → 259.3 were selected for F, F-d_4_, DHEA, DHEA-^13^C_3_, Δ5-diol, 3βAdiol, and Δ5-diol-d_4_, respectively.

##### 7α-OH-DHEA

The residue was treated with 0.12 mL reaction mixture (20 mg *O*-ethylhydroxyl-ammonium chloride in 1 mL acetonitrile/distilled water (60:40, v/v)) for 30 min at 60 °C. After derivatization, the reaction mixture was subjected to LC-MS/MS. For quantitation, the *m/z* transitions 348.2 → 270.0 and 351.1 → 273.2 were selected for 7α-OH-DHEA and 7α-OH-DHEA-d_2_, respectively.

##### E2

The residue was reacted with the reaction mixture (50 μL pentafluoropyridine, 40 μL 1 M sodium hydroxide solution, 100 μL acetonitrile, and 20 μL ethanol) for 20 min at room temperature. After the reaction, the mixture was evaporated to dryness. Distilled water was added to the residue, and derivatized E2 was extracted with hexane. After the hexane layer was evaporated to dryness, the extracted E2 underwent further derivatization. The residue was reacted with 50 μL of reagent mixture (MNBA, 80 mg; FA, 40 mg; DMAP, 20 mg in 1 mL acetonitrile) and 10 μL TEA, and then left at room temperature for 30 min. After derivatization, the reaction mixture was cleaned with an InterSep SI cartridge. After purification, the extract was evaporated to dryness, the residue was dissolved in acetonitrile/distilled water (80:20, v/v), and the solution was subjected to LC-MS/MS. For quantitation, the *m/z* transitions 583.3 → 308.1 and 587.2 → 311.8 were selected for E2 and E2-^13^C_4_, respectively.

### Data Analysis and Statistics

Analyses were performed using StatMate IV for Windows (ATOMS, Tokyo, Japan). All statistical tests were two-sided, and significance was defined as p < 0.05. Based on the preliminary nature of the study, calculation of sample size based on specific hypotheses was not appropriate.

Group mean comparisons of the serum and salivary levels of each steroid were performed using Kruskal–Wallis H statistics and chi-square distributions. If Kruskal–Wallis H tests were significant, differences between pairs of groups were evaluated by multiple comparisons using Dunn’s tests. Spearman correlation coefficients were used to evaluate the correlation between the serum and salivary levels of each steroid. Missing data were excluded from analyses.

BMI was compared between groups using one-way analysis of variance (ANOVA) after homogeneity of variance was assessed by Bartlett’s test. Mean body fat percentages and abdominal circumferences of control and depression groups of each sex were compared using variance ratio F and Welch’s tests after homogeneity of variance was assessed using F tests.

Some values of E2, 7α-OH-DHEA, Δ5-diol, and 3βAdiol were under the limit of quantitation. The lower limits of quantitation were as follows: E2, 0.03 pg/assay; DHEA, 7α-OH-DHEA and 3βAdiol, 2 pg/assay; Δ5-diol, 1 pg/assay; and F, 50 pg/assay.

## Additional Information

**How to cite this article**: Kobayashi, M. *et al*. Sex differences in the serum level of endogenous ligands for estrogen receptor β in the elderly population. *Sci. Rep.* **6**, 25878; doi: 10.1038/srep25878 (2016).

## Supplementary Material

Supplementary Information

## Figures and Tables

**Figure 1:**
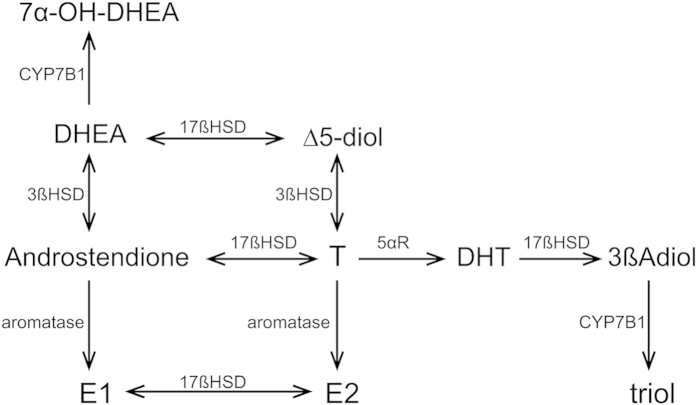
DHEA is a precursor of endogenous ligands for ERβ. The main pathway of 3βAdiol production is from testosterone (T). Δ^4^-3-ketosteroid-5α-reductase (5αR) converts T to 5α-dihydrotestosterone (DHT). 17β-hydroxysteroid dehydrogenase type 7 (17βHSD VII) converts DHT to 3βAdiol. 3βAdiol is metabolized by cytochrome P450, family 7, subfamily B, polypeptide 1 (CYP7B1) to 5α-androstane-3β, 7α, 17β-triol (triol). T is synthesized from Δ5-diol (Androstenediol) via 3βHSD, and Δ5-diol is from DHEA (dehydroepiandrosterone) via 17βHSD.

**Table 1:** Clinical data for subjects.

	Male controls (n = 12)	Female controls (n = 16)	Male depression (n = 10)	Female depression (n = 13)	*Kruskal–Wallis test*	*Dunn’s test*
Age	65.7 ± 5.7	60.6 ± 2.3	67.4 ± 7.0	68.8 ± 6.7	H = 46.65, df = 3, p = 0.006	F/C vs. F/D (p < 0.01)
Height (cm)	166.2 ± 5.1	155.9 ± 4.7	166.4 ± 6.8	152.3 ± 4.6		
Weight (kg)	70.3 ± 9.5	56.6 ± 7.7	66.9 ± 7.0	53.0 ± 5.5		
BMI	25.4 ± 3.2	23.3 ± 3.1	24.2 ± 2.9	22.9 ± 2.3	H = 46.62, df = 3, p = 0.193	
Percentage of Body Fat (%)	24.3 ± 4.3	32.5 ± 5.1	24.0 ± 3.7	32.9 ± 4.2	H = 16.65, df = 3, p < 0.001	M/C vs. F/C (p < 0.01)M/C vs. F/D (p < 0.01)F/C vs. M/D (p < 0.01)M/D vs. F/D (p < 0.01)
Abdominal Circumference (cm)	88.5 ± 6.0	82.2 ± 10.0	84.3 ± 5.4	78.3 ± 7.3	H = 46.62, df = 3, p = 4.18 × 10^−10^	M/C vs. F/D (p < 0.001)
HAM-D17	2.0 ± 2.1	2.0 ± 1.9	4.6 ± 2.3	3.4 ± 2.0	H = 4.66, df = 3, p = 0.020	F/C vs. M/D (p < 0.001)
BDI-II	4.1 ± 2.4	6.0 ± 4.3	6.0 ± 4.1	6.8 ± 3.9	H = 2.92, df = 3, p = 0.481	
MMSE	29.6 ± 0.8	29.7 ± 0.5	28.2 ± 1.5	28.1 ± 1.9	H = 3.89, df = 3, p = 0.274	
FAB	16.7 ± 1.2	17.1 ± 0.7	16.4 ± 0.7	15.9 ± 1.5	H = 6.73, df = 3, p = 0.094	
CDR	0	0	0	0		

The values are shown as mean ± standard deviation. Four group mean comparisons of anthropometric measurements and psychiatric inventories were performed. *Abbreviations: BDI-II,* Beck Depression Inventory II; *BMI,* Body Mass Index; *CDR,* Clinical Dementia Rating; *FAB,* Frontal Assessment Battery; *HAM-D,* Hamilton Rating Scale for Depression 17 items; *MMSE,* Mini Mental State Examination; *F/C,* female controls; *F/D,* female depression group; *M/C,* male controls; *M/D,* male depression group.

**Table 2:** Serum steroid levels.

	Male Controls (n = 12)	Female Controls (n = 16)	Male Depression (n = 10)	Female Depression (n = 13)	*Kruskal–Wallis test*	*Dunn’s test*
E2 (pg/mL)	**15.87** ± **5.56**	**2.24** ± **1.39**	**21.23** ± **8.20**	**2.08** ± **1.36**	**H** = **18.80df** = **3p** = **1.87 × 10**^−**7**^	**M/C vs. F/C (p** < **0.01)**
						**M/D vs. F/D (p** < **0.001)**
DHEA (ng/mL)	1.87 ± 0.65	2.24 ± 0.83	1.36 ± 0.45	1.50 ± 1.29	H = 9.18df = 3p = 0.003	F/C vs. F/D (p < 0.001)
7α-OH-DHEA (pg/mL)	147.92 ± 67.59	133.31 ± 40.65	113.06 ± 54.28	116.87 ± 83.76	H = 2.81df = 3p = 0.203	
3β Adiol (pg/mL)	**25.08** ± **20.06**	**5.69** ± **7.31**	**22.23** ± **10.22**	**3.52** ± **3.41**	**H** = **20.03df** = **3p** = **1.11 × 10**^−**6**^	**M/C vs. F/C (p** < **0.01)M/D vs. F/D (p** < **0.001)**
Δ5-diol (ng/mL)	**0.50** ± **0.31**	**0.23** ± **0.07**	**0.47** ± **0.18**	**0.16** ± **0.13**	**H** = **19.15df** = **3p** = **1.67** × **10**^−**5**^	**M/C vs. F/C (p** < **0.001)M/D vs. F/D (p** < **0.05)**
F (ng/mL)	103.30 ± 40.99	82.22 ± 26.87	106.96 ± 32.10	72.08 ± 19.59	H = 6.19df = 3p = 0.025	**M/D vs. F/D (p** < **0.05)**

The values are shown as mean ± standard deviation. Four group mean comparisons of serum levels of each steroid were performed. *Abbreviations: 3*β *Adiol*, 5α-androstane-3β,17β-diol; *7*α*-OH-DHEA,* 7α-hydroxydehydroepiandrosterone; Δ*5-diol,* Androstenediol; *DHEA* dehydroepiandrosterone; *E2,* 17β-estradiol; *F,* cortisol; *F/C,* female controls; *F/D,* female depression group; *M/C,* male controls; *M/D,* male depression group.

**Table 3:** Correlation between serum and salivary concentrations of each steroid.

E2	DHEA	7α-OH-DHEA	3β Adiol	Δ5-diol	F
rs = −0.274	rs = −0.564	rs = −0.546	rs = −0.351	rs = −0.267	rs = −0.438
n.s.	p < 0.001	p < 0.001	p < 0.05	n.s.	p < 0.01

Spearman correlation coefficients were used to evaluate the correlation between the serum and the salivary concentrations of each steroid. *Abbreviations: 3*β*Adiol,* 5α-androstane-3β,17β-diol; *7*α*-OH-DHEA,* 7α-hydroxydehydroepiandrosterone; Δ*5-diol,* Androstenediol; *DHEA,* dehydroepiandrosterone; *E2,* 17β-estradiol; *F,* cortisol.
