# Supplementary material for: Sex differences in the serum level of endogenous ligands for estrogen receptor β in the elderly population
Source: Sci Rep. 2016 May 11;6:25878. doi: 10.1038/srep25878 (PMC4863166; doi:10.1038/srep25878)
Supplement: Supplementary Information [file srep25878-s1.pdf]

Supplementary information

Sex differences in the serum level of endogenous ligands for estrogen receptor β in the elderly population

Miyuki KOBAYASHI[1, 2], Nobuhiro SUGIYAMA[1, *], Daimei SASAYAMA[1], Hidehiko SASAMOTO[3], Yoshimichi MIYASHIRO[3], Kunimasa ARIMA[2], Shinsuke WASHIZUKA[1]

[1]Department of Psychiatry, Shinshu University School of Medicine, 3-1-1 Asahi, Matsumoto, Nagano 390-8621, Japan; [2]Department of Psychiatry, National Hospital Organization Komoro Kogen Hospital, 4598 Ko, Komoro, Nagano 384-8540, Japan; [3]ASKA Pharmaceutical Medical Co., Ltd. 5-36-1 Shimosakunobe, Kawasaki Takatsu-ku, Kanagawa 213-8522, Japan;

**Corresponding author and person to whom reprint requests should be addressed:*

Nobuhiro Sugiyama, MD, PhD

Supplementary Information Figure S1

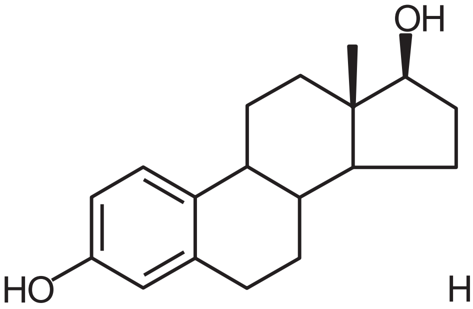

17ß-Estradiol
(E2)

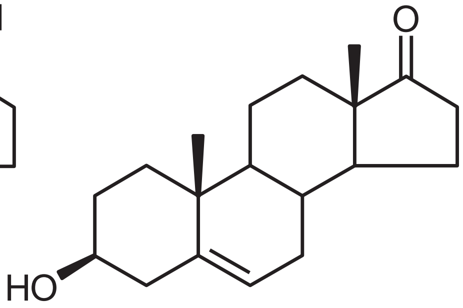

Dehydroepiandrosterone
(DHEA)

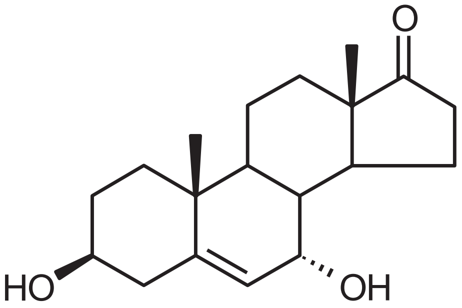

7α-hydroxydehydroepiandrosterone
(7α-OH-DHEA)

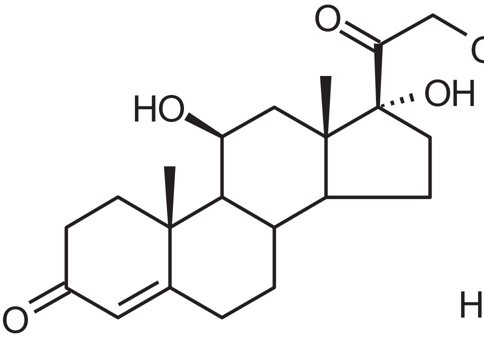

Cortisol
(F)

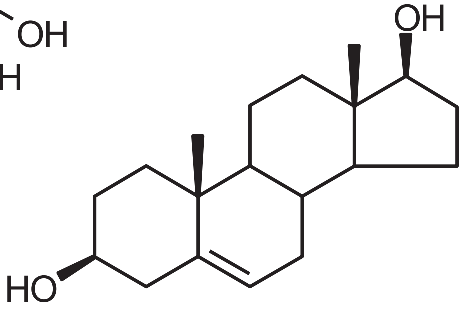

Androstenediol
(∆5-diol)

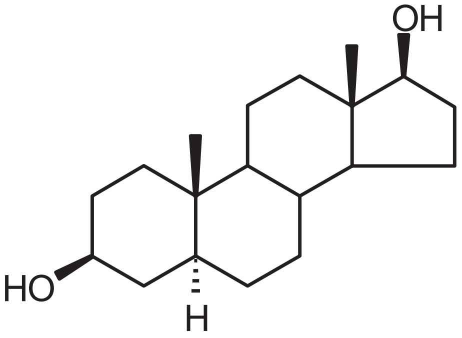

5α-androstane-3ß, 17ß-diol
(3ßAdiol)

Figure S1. Chemical structures of the steroids of interest

Supplementary Information Figure S2

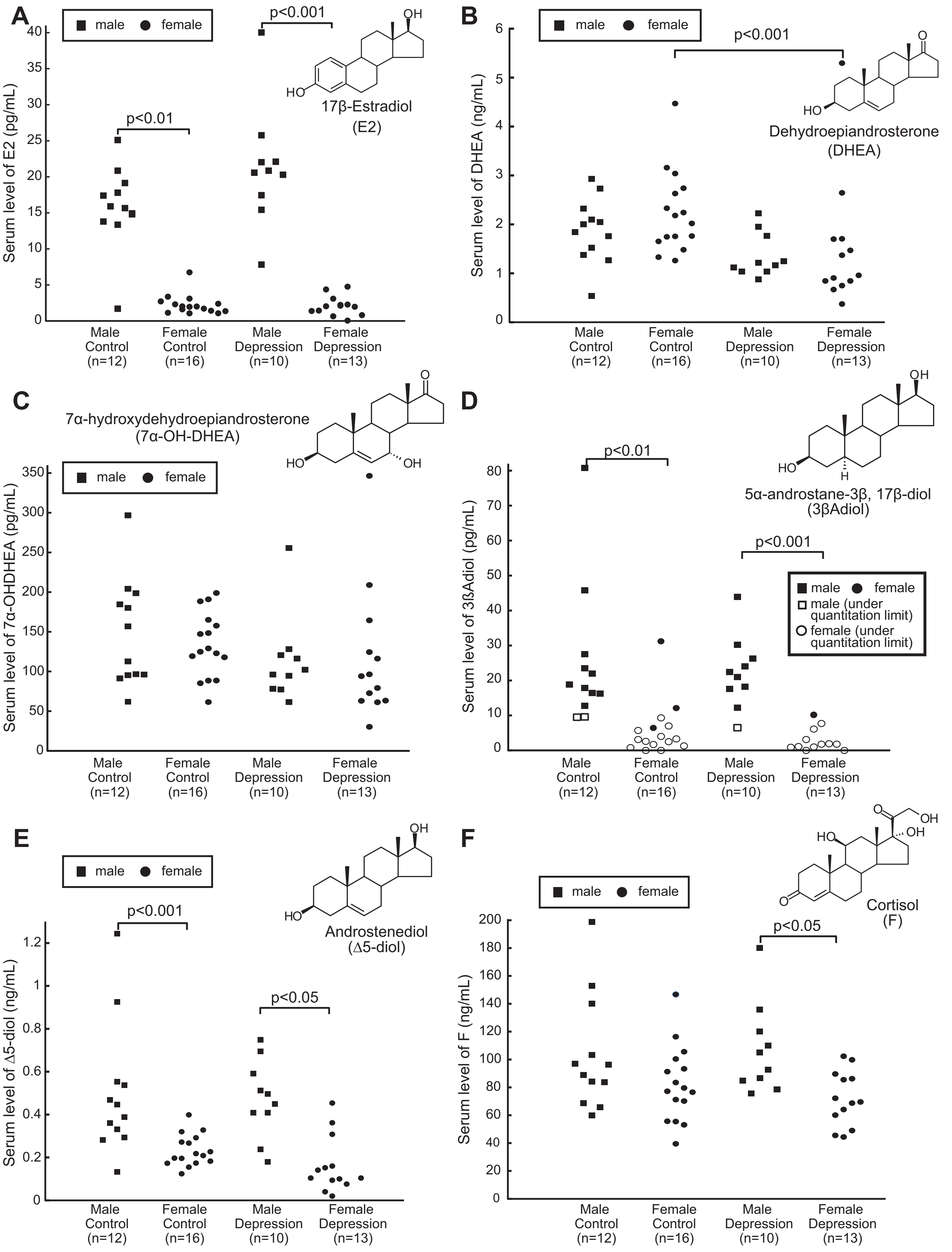

Figure S2. **Serum Levels of Steroids**

Supplementary Information Figure S3

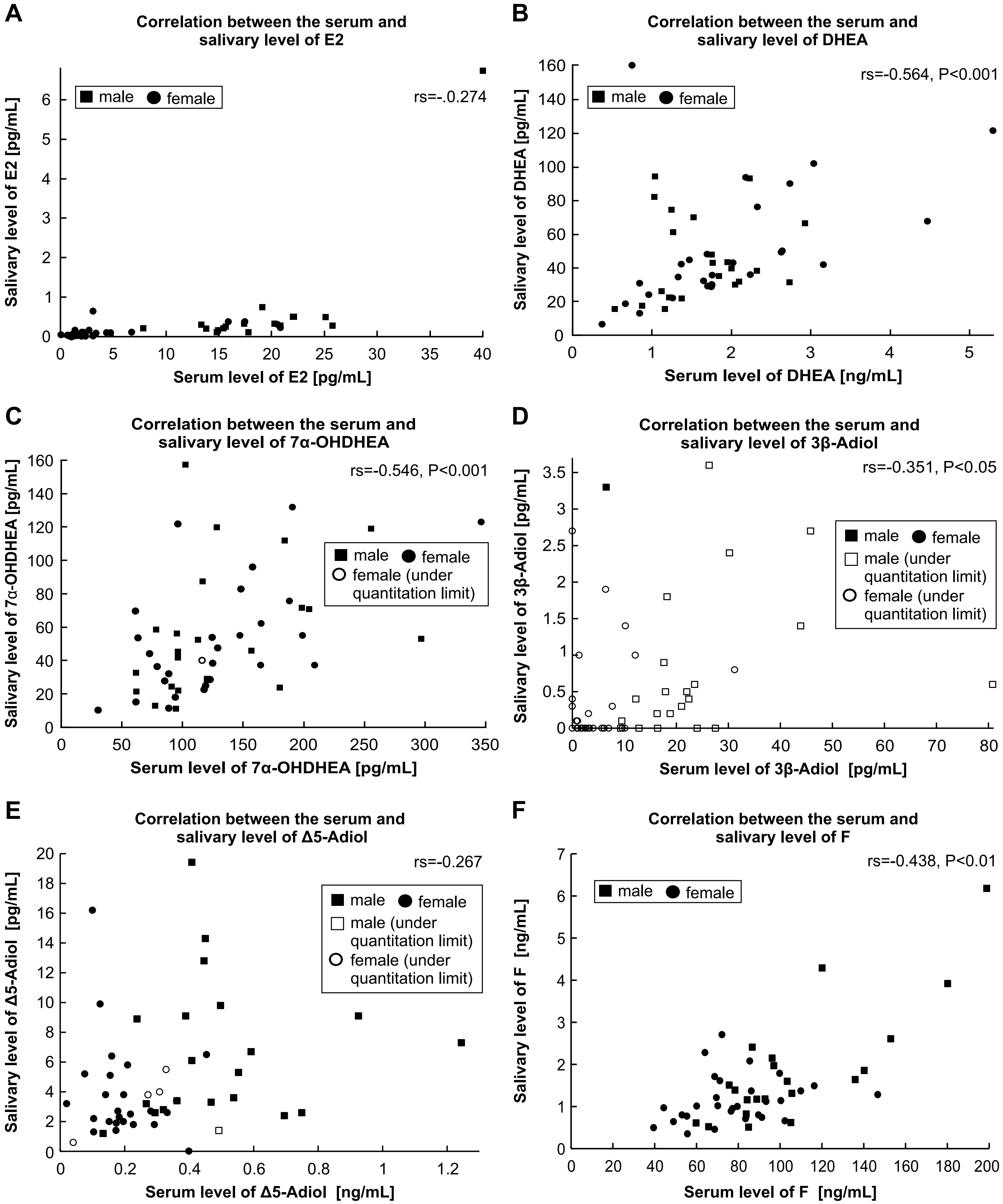

Figure S3. Correlation between the Serum and Salivary Levels of Each Steroid
